# Exploring Gendered Pathways: Understanding the Connection Between Female Substance Misuse and Offending Behavior

**DOI:** 10.1002/jcop.70062

**Published:** 2025-12-02

**Authors:** Dikoetje Frederick Monyepao

**Affiliations:** ^1^ University of South Africa Pretoria South Africa

**Keywords:** criminal justice system, gender, incarcerated women, life adversities, prison, substance misuse, victimization

## Abstract

This study, grounded in community psychology, explored the lived experiences of incarcerated women in Gauteng, South Africa, focusing on the links between substance use, victimization, and adverse childhood experiences. Semi‐structured interviews were conducted individually with 29 women incarcerated in 2 correctional centers in South Africa: Kgoši Mampuru II and Johannesburg Correctional Centre. Interviews explored participants' family backgrounds, experiences with substance use, and involvement with the criminal justice system. The analysis highlighted that participants frequently described substance use as a way of coping with trauma and adversity. This study highlights the need for community‐based interventions that address the social determinants of health and empower women to overcome adversity. Implications for trauma‐informed care, community support, and social justice advocacy within a community psychology framework are discussed. Gender‐responsive approaches in substance use programs for both practitioners and policymakers are also referenced.

## 1 Introduction

Community psychology (CP) emphasizes the interconnectedness of individuals and their environments, recognizing that social problems are rooted in complex interactions between personal, social, and structural factors (Watson‐Thompson et al. 2021). The incarceration of women, therefore, is not simply an individual issue but a community concern with far‐reaching consequences for families, social networks, and community well‐being. Women constitute nearly half of the world population, women's contributions are pivotal to its foundations. Their contributions are essential to the progress, development, and well‐being of communities globally. However, the incarceration of women often results in emotional and economic hardship for their families, particularly children, creating ripple effects that destabilize communities and exacerbate social issues (Condry and Minson 2021, 4; Minson 2019). As the incarceration of women continues to rise (Tubex and Gately 2025), it becomes crucial to examine the unique challenges they face, particularly in relation to substance use and involvement with the criminal justice system (CJS), to mitigate further individual and societal harm. Aligned with the values of community psychology, this study seeks to understand the experiences of women involved with the CJS, with the goal of informing community‐based interventions that promote empowerment, social justice, and, ultimately, prevention of further involvement with the CJS.

Historically and contemporarily, research on the relationship between substance use and involvement with the CJS has predominantly focused on men (Contreras and Hipp 2020; MacGregor 2000; Newcomb et al. 2001; Rolando et al. 2021; White et al. 1985). This male focus often obscures the complex, gender‐specific factors influencing women's experiences with substance use and involvement with the CJS. Additionally, mainstream psychology tends to overemphasize the individual's pathology rather than recognizing the broader social, political, and economic circumstances that contribute to substance‐related harm (Riemer et al. 2020). Instead of locating drug use and its effects solely within individuals, a more community‐based, locally informed approach can acknowledge systemic inequalities, cultural differences, and collective responsibilities.

Women's experiences with substance use and involvement with the CJS are shaped by interconnected factors, including trauma, family dynamics, peer influence, economic hardship, mental health challenges, and structural barriers (Ahmed et al. 2023, 5–8; Boppre and Boyer 2021, 432; Green et al. 2019, 8). Understanding these intricate dynamics is fundamental to developing effective, gender‐responsive interventions tailored to the needs of women facing these challenges (Anderson et al. 2019, 26; Henry 2020). Trauma, family dynamics, peer influence, economic hardship, mental health challenges, and structural barriers align closely with Bronfenbrenner's ecological systems theory, where each category operates at a particular ecological level (Bronfenbrenner 2000). At the microsystem level, immediate influences such as family relationships or personal coping strategies shape individual behaviors. At the mesosystem level, the interactions between small settings such as families, peer groups, and service providers influence experiences and outcomes. At the exosystem level, factors like employment opportunities or local support networks indirectly shape women's experiences through policies and structures that affect daily living. At the macrosystem level, broad cultural norms, social values, and large‐scale governance create overarching conditions that either promote or inhibit equitable support.

This study sought to explore the multifaceted experiences that contribute to some women's substance use and involvement with the CJS, shedding light on the complexities of these experiences and emphasizing the necessity of gender‐responsive approaches. By addressing the underlying factors associated with women's substance use and involvement with the CJS, gender‐sensitive interventions can empower women to make positive changes and build fulfilling lives (Meyer et al. 2019, 10; Migliorini et al. 2020). To foster these approaches, it is essential to deepen our understanding of the factors that contribute to women's substance use and involvement with the CJS. This paper reviews the existing literature on the link between substance use and CJS involvement, identifies its limitations, and highlights critical gaps. It proceeds to outline the study's methodology and ethical considerations, presents the insights and discussions, and concludes with recommendations informed by the insights gathered.

### 1.1 Gendered Perspectives on Substance‐Related Crime

The relationship between drug use and involvement with the CJS is complex, shaped by intersecting social and structural factors, particularly in the lives of women (Liu et al. 2021). Research into women's experiences with the CJS within this field remains limited, partly due to the smaller population of women in contact with the CJS (Ahmed et al. 2023, 2; Estrada et al. 2019, 139; Kruttschnitt et al. 2019, 485). Dedicated research into women's experiences with the CJS is essential, as it informs the development of policies and interventions tailored to gender‐specific needs. Recent decades have seen an increase in the number of women in contact with the CJS (Dyvik 2023; Fair and Roy 2022; Renal Reform International 2022).

Traditional criminological theories, often critiqued by CP for their individualistic and pathologizing perspectives (Riemer et al. 2020), frequently explained women's involvement with the CJS through gendered stereotypes. These theories often associated women's interactions with the CJS with actions such as shoplifting, prostitution, and other behaviors labeled as “sexually deviant” (Campbell 1981). Early researchers attributed women's lower participation in street‐level offenses to societal restrictions on their public presence. Conversely, men's involvement with the CJS was seen as an expression of cultural aspirations, peer approval, and defiance of societal norms (Farnworth and Leiber 1989). However, trends in women's contact with the CJS have shifted significantly over time (Ayang et al. 2022; Estrada et al. 2019; Yerger et al. 2022). Early criminological perspectives undervalued women's involvement with the CJS, deeming it negligible due to the perceived lower numbers of women in contact with the CJS (Bowker et al. 1978; Steffensmeier and Streifel 1992; Steffensmeier and Haynie 2000).

In South Africa, the legacy of apartheid has created enduring social and economic inequalities, which are central to community psychology's focus on social justice (Malherbe and Dlamini 2020; Ratele and Malherbe 2022). These inequalities are reflected in insights by Dada et al. (2018), who documented changes in women's substance use patterns in the Western Cape Province, including a rise in the number of women seeking treatment for substance misuse between 2000 and 2013. No reason was specified for this increase, and it is not the endeavor of this study to address treatment‐seeking behavior. Their study highlighted the prevalence of alcohol, methamphetamine, and polysubstance misuse among women, which was associated with significant psychological and social challenges. However, the study lacked contextual analysis of the relationship between substance misuse and involvement with the CJS. South African and African literature on the link between substance use and CJS involvement (Malaka and Da Rocha‐Silva 2008; Mokwena and Morojele 2014; Chetty 2015; Dintwe 2017; Olawole‐Isaac et al. 2018) generally assumes a causal relationship, aligning with punitive approaches. These studies often include contact with the CJS as a secondary aspect rather than as a central focus, limiting their contextual relevance. This is particularly problematic as it fails to consider the complex interplay of factors that contribute to both substance misuse and criminal behavior, such as poverty, trauma, and gender‐based violence.

This is especially relevant in the South African context, where high rates of gender‐based violence (Jewkes 2022; Oeschger et al. 2024), poverty (Cowling 2024), and historical trauma related to apartheid (Ratele and Malherbe 2022) intersect to create unique vulnerabilities for women. Furthermore, research has shown the link between trauma and substance misuse as a coping mechanism, particularly in women (Najavits 2000; Ndungu et al. 2020). A more nuanced understanding is needed that moves beyond anecdotal assumptions of causality and explores the social and structural contexts within which these issues occur. Community psychology, with its emphasis on context and social justice, provides an effective framework to analyze these dynamics. Factors such as the legacy of apartheid, ongoing poverty, and pervasive gender‐based violence represent forms of structural violence. This concept is used to describe the ways in which social structures and systems systematically harm or disadvantage certain groups (Galtung 1969). These structural inequities contribute to the marginalization of women, creating environments that exacerbate substance misuse and increase the likelihood of CJS involvement (Spector et al. 2021). By addressing these inequities, CP seeks to foster community well‐being and social change.

Additionally, the liberation psychology approach emphasizes the importance of understanding historical and systemic oppression, particularly in postcolonial contexts like South Africa (Martín‐Baró 1994). The legacy of apartheid, with its entrenched racial and gender inequalities, continues to influence the economic and social realities faced by many women, further perpetuating cycles of poverty, trauma, and substance misuse (Fernandez 2020). Liberation psychology calls for the transformation of these oppressive systems, focusing on collective empowerment and advocacy for systemic change (Burton and Guzzo 2020). In line with these principles, this study seeks to contribute to the field of CP by exploring the multifaceted experiences that contribute to some women's substance misuse and involvement with the CJS. By using a phenomenological approach, it aims to provide contextualized insights into these women's lived experiences, highlighting the connections of structural violence, trauma, and substance use. This understanding can inform the development of more effective, community‐based, and empowering interventions aligned with community psychology's commitment to addressing social inequalities and promoting community well‐being.

## 2 Methods

Given the exploratory nature of this study, a qualitative approach was employed. This approach complements inductive research (Corley et al. 2021, 163) and is well‐suited for examining relationships among complex concepts (Agazu et al. 2022, 1500). In this study, the focus was on understanding the interplay between substance use and involvement with the CJS. The qualitative approach allowed for an in‐depth exploration of the meanings and experiences associated with this relationship, with a specific focus on gender. To further explore these lived experiences, a phenomenological research design was adopted. Phenomenological studies explore individuals' lived experiences to understand the essence of a phenomenon, focusing on subjective perceptions rather than objective facts (Williams 2021, 369). These studies typically involve recruiting participants who have direct experience with the phenomenon and data collection using open‐ended, semi‐structured interviews, reflective journals, or focus groups. Questions are designed to elicit detailed personal accounts, often beginning with prompts such as, “Can you describe your experience in detail?” (Van Manen and Van Manen 2021, 1074).

The strengths of phenomenological studies lie in their ability to provide rich, nuanced insights into complex or under‐researched phenomena. They prioritize capturing participants' subjective meanings, offering a depth of understanding that other methods may overlook. The design informs the research process by shaping interview questions to focus on lived experiences, employing purposeful sampling to select participants, and requiring the researcher to bracket their own biases to center participants' perspectives (Rasid et al. 2021). Data analysis in phenomenological research involves inductively identifying significant statements, extracting themes, and synthesizing these into a description of the phenomenon's essence. A common approach is Giorgi's (2020) method (used in this study), which emphasizes thematic exploration while prioritizing the preservation of participants' voices and avoiding the influence of preexisting theories or frameworks.

### 2.1 Participants

This study focused on women who were incarcerated and who had a history of substance use prior to their incarceration. Participants were required to be 18 years or older, the legal age of adulthood in South Africa. The youngest participant was 21 years old, and the oldest was 52. The majority (62.1%) of participants were unemployed prior to incarceration, while those who were employed primarily engaged in informal work. Only 20.7% of participants held formal employment across various sectors. Informally employed participants relied on temporary and irregular jobs, resulting in unstable income. In terms of educational attainment, 72.4% of participants did not complete secondary schooling, with 27.6% leaving school during primary education. Only 17.2% of participants completed their matriculation, and 10.3% pursued higher or tertiary education post‐matriculation. Regarding racial demographics, the participants comprised 15 Black (51.7%), 4 White (13.8%), 9 Mixed race (31%), and 1 Asian (3.4%). These participants were considered most likely to provide relevant information for the research aims. A nonprobability sampling technique, specifically snowball sampling, was used to recruit participants (Ellis 2021, 130). This method was chosen due to the challenges in accessing this population. Because the Department of Correctional Services (DCS) does not keep records categorizing substance use in relation to specific offenses, snowball sampling—where existing participants were asked to connect the researcher with other potential participants who met the study criteria—was the most effective recruitment strategy.

Table 1 provides the types of offenses the women were imprisoned for. The terminology in the table refers: contact crime refers to offenses involving physical harm or threats to persons (e.g., assault and homicide). Other serious crime includes noncontact but high‐impact offenses such as fraud and corruption. Property‐related crime involves unlawful appropriation or damage to property (e.g., burglary, theft, arson).

**Table 1:** Types of offenses for which serving a prison sentence for.

Crime category	Number of women
Contact crime	17
Other serious crime	1
Property‐related crime	11

### 2.2 Materials

In the undertaking to further understand the relationship between female substance use and criminal behavior, women who are incarcerated were sought from two correctional centers. Semi‐structured interviews were conducted face‐to‐face, where questions related to family history, criminal history, and substance use history were asked. Examples of questions include: “How did you start using drugs?”, “From your perspective, what do you think led you to drug use and criminal behavior?”, and “Have you engaged in illegal activities in order to get access to drugs?” Demographic information of the participants was also captured. Permission for the audio recording of the interviews was also granted by both the participants and the correctional facilities. Coupled with field notes that recorded nonverbal cues, transcriptions were made promptly after the interview sessions by the interviewer/researcher. Thereafter, the transcripts were thematically analyzed following the steps outlined by Thompson (2022, 1412–1416) for thematic analysis. The software Atlas.ti was used for the coding process. This study allowed the researcher to highlight texts and code them to specific nodes with labels such as “substance use,” “type of crime,” “experienced trauma,” “upbringing,” etc. These nodes were further filtered down into themes, which formed part of the data.

### 2.3 Study Context and Data Collection Procedures

This study was conducted in Gauteng Province, South Africa, a predominantly urbanized region marked by significant socioeconomic disparities and the enduring spatial legacies of apartheid. The two correctional centers were Johannesburg Correctional Centre, located in Johannesburg, and Kgoši Mampuru II Correctional Centre, located in Pretoria. Similar to other parts of South Africa, Johannesburg and Pretoria experience high unemployment rates, poverty, and high crime rates. The apartheid‐era spatial planning further contributed to segregation, limited access to resources, and overcrowding, which continue to affect the social and economic conditions of residents. Both facilities have dedicated female sections alongside their male divisions, addressing the needs of women in the correctional system. Female correctional facilities in South Africa house inmates based on available space, not district, resulting in facilities accommodating women from diverse demographics across the country. Of the 91 facilities with female sections, 2 were selected for this study due to their accessibility for the researcher, which was crucial for logistical reasons such as travel distance and related costs.

A total of 29 women participated in the study. Data collection began at Johannesburg Correctional Centre, where interviews were conducted with 21 participants. Due to the significant distance between Johannesburg Correctional Centre and Kgoši Mampuru II Correctional Centre, data collection was carried out separately at each facility. Once data collection at Johannesburg Correctional Centre was completed, the researcher proceeded to Kgoši Mampuru II Correctional Centre, where the remaining eight participants were interviewed. The interviews were conducted by the researcher/author, who has a PhD in Criminology and is a seasoned researcher in qualitative research strategies. The interviews followed a semi‐structured format, using an interview guide with open‐ended questions to explore participants' experiences. The interviews lasted an average of 1 h and took place within the respective correctional centers. The interviews were conducted in a room that was within eyesight of the correctional centers' officials/guards. For safety purposes, the interview room's door was kept open at all times at both correctional centers.

Recruitment at the sites concluded when no further eligible participants could be identified through the snowball sampling method. The final sample size of 29 was determined by data saturation, the point at which no new substantive insights were emerging from the interviews. The semi‐structured interview format allowed for flexibility in questioning; questions were adapted as needed during the interviews to facilitate follow‐up inquiries, clarifications, and deeper exploration of participants' experiences. The interview guide included questions that garnered information on “history of substance use,” “history of commission of crimes,” and “family background from childhood to adulthood.”

### 2.4 Data Analysis

This study utilized inductive thematic analysis, which implies that the process of coding did not fit preexisting themes or the researcher's preconceptions. Inductive thematic analysis is heavily data‐driven as opposed to deductive thematic analysis, which is theory‐driven. The inductive approach entails that the theme identified is linked to the data and bears no connection to the questions posed to the research subjects (Braun and Clarke 2023, 2). It must be acknowledged that pure induction could not be achieved because the analysis was influenced by the coders' academic training, experience, and epistemology. To cater for this potential bias, the coders stayed as close as possible to the meanings in the data, as advised by Morgan (2022, 2081). This means that the data were presented as is and not as the coders presumed it to be.

Any discrepancies in coding were resolved through discussion and consensus between the two coders. After reaching a consensus on the coding, the primary researcher then synthesized the coded data and developed the final thematic structure, ensuring alignment with the study's aims and research questions. While the final thematic structure was determined by the primary researcher, the collaborative coding process provided valuable insights and ensured that the themes were grounded in the data. This consensus, or intercoder agreement, increases the trustworthiness and dependability of the data sets (Cascio et al. 2019, 1).

The data analysis was further guided by a phenomenological approach, focusing on understanding the essence of the participants' lived experiences. This involved bracketing the researcher's preconceptions and focusing on the participants' descriptions of their experiences, identifying common themes and patterns across the interviews.

### 2.5 Strategies to Enhance Rigor

To ensure trustworthiness, the research process adhered to four key criteria: credibility, transferability, dependability, and confirmability. Credibility, the alignment between participants' perspectives and their representation by the researcher (Nassaji 2020, 428), was established through adherence to established qualitative research procedures (Rose and Johnson 2020, 4). Member checking, which involves sharing insights with participants for verification, was not feasible in this study as the researcher had only one opportunity to interact with each participant. However, the researcher kept an audit trail and engaged in peer debriefing to ensure credibility. Transferability, the extent to which meanings from one context can resonate in other contexts (Hays and McKibben 2021, 178), was addressed by providing detailed descriptions of the participants, research settings, and context, as stated above in Section 2. Dependability, referring to the stability of data and research processes (Rose and Johnson 2020, 5), was ensured through the maintenance of a detailed process log documenting methodological and theoretical decisions throughout the study. Confirmability, reflecting the researcher's ability to minimize personal bias and maintain objectivity (Muzari et al. 2022, 18), was supported by meeting the preceding three criteria and by explicitly stating the rationale for all theoretical, methodological, and analytical choices.

### 2.6 Ethical Considerations

As a matter of good ethical research practice, this study was approved by the University of South Africa's (UNISA) College of Law (CLAW) Research Ethics Committee for ethical clearance before it could commence. Additionally, the study was approved by the DCS Research Committee before collecting data within the correctional facilities. For the semi‐structured interviews to be conducted, informed consent had to be granted by the participants (Husband 2020, 2). To assure the authenticity of the consent, participants were requested to sign an informed consent form before participating in the research. It was stipulated in the consent form and further explained that participation in the study was completely voluntary. As incarcerated women with experiences of substance misuse, these participants are considered a vulnerable population. First, because they are incarcerated and, second, because they may also have misused substances. Therefore, to obtain legitimate consent, the researcher provided an overview of the research as well as the data collection process to the participants before the interviews. Furthermore, the potential discomforts, risks, and expected benefits were explained. The researcher was transparent (avoided any forms of deception) with the participants and further explained that there were no direct benefits to participating in the study. The participants were also notified that they were at liberty not to divulge information they were not comfortable with and could withdraw from the research at any point.

In cases where the researcher noted that participants were uncomfortable, the researcher paused during the interview and asked the participants if they wanted to continue. Participants were also informed that the social workers in the correctional facilities were available for debriefing should they require any. In all instances during the semi‐structured interviewing process, social and cultural sensitivity was practiced concerning the age, gender, culture, religion, and social class of the participants. The interviews were conducted in a secure and safe space where participants could feel free to share their lived experiences. As a safety measure, in accordance with correctional facility protocols, a DCS official was stationed outside the interview room, and the door remained open throughout each interview. This protocol, which prohibits any male from being alone with female inmates, was implemented to ensure the safety and security of both the researcher and participants. This could have affected the quality of data collected, as well as the anonymity of participants. However, most participants confirmed that they felt free to continue with the semi‐structured interviews despite the safety Protocol.

### 2.7 Emergent Themes

Analysis of the data, particularly the co‐occurrence of the codes “experiences of trauma” and “substance use,” highlighted victimization as a prominent theme. The following are the emergent themes: *substance use as a coping mechanism for victimization* and s*ubstance use related to other adverse life experiences*. “Early victimization” refers to experiences of victimization that occurred during childhood. “Adverse life experiences” encompasses negative events that did not involve direct victimization but nonetheless contributed to participants' substance use. These experiences included the death of a close caregiver, a lack of belonging within family or community, exposure to caregivers' substance use during childhood, and introduction to substances through peers or environmental influences. Given the study's focus on the relationship between substance use and involvement with the CJS among women, a description of factors contributing to their contact with the CJS is also provided. Pseudonyms were used for all participant names in the direct quotes and were randomly generated using an online Tool.

#### 2.7.1 Substance use as a Coping Mechanism for Victimization

Many of the women in this study experienced significant victimization early in life, with the majority reporting experiences of sexual abuse, including rape, perpetrated by both individuals known to them and strangers. Others experienced intimate partner violence, including physical violence, in early adulthood. Participants shared their experiences of abuse and described how they subsequently used substances as a coping mechanism.

…there were these other guys sitting by a container, and they had a gun. When I approached them, they took out their gun, and I felt like I was going to lose my life. […] They took me to this other yard, and they forced me to sleep with them… They raped me. […] I just concluded by keeping this (rape incident) in my heart. This is the thing that made me to always want to be under the influence… I was 17 going on to 18.

Rachel

Rachel's account reveals how sexual trauma at a formative stage contributed to her dependence on substances as a means of emotional numbing. Her words highlight how unresolved trauma and the absence of external support can lead to internalized suffering and reliance on self‐soothing through intoxication. Within a constructivist frame, Rachel constructs meaning from her experience through survival, not pathology.

Where my grandmother worked, she used to stay there, and only come back at the end of the month. I was alone with my step‐grandfather, and when I was 14, he started to rape me. […] Then I went to my friend's place, and we were smoking, drinking alcohol and taking drugs.

Sarah

Sarah's reflection illustrates how abuse within trusted family spaces prompted early exposure to substance use as a coping strategy. Her narrative captures both emotional isolation and the search for temporary relief in peer contexts, reflecting how early victimization shapes pathways into risky environments.

I just found the guy and here was the money and it was all bloomy, not knowing that this is going to be my child's father one day and we will end up getting married one day. But with all this came abuse, physical abuse, psychological abuse, and emotional abuse. With this, I started with the rocks (crack cocaine) again.

Megan

Megan's description connects relational violence with recurring substance use. Her narrative shows how cycles of dependency and abuse intertwine, where crack cocaine use symbolizes both resistance to and entrapment within abusive dynamics.

These experiences of victimization often precipitated substance use. For many participants, reminders of past sexual abuse or renewed partner abuse rekindled psychological distress, leading to the use of substances as a coping mechanism.

At home, I used to sit with this cousin (sexual abuse perpetrator), and then my anger would go up. Then I would go out. I couldn't face him and I would constantly be asking why did he do this to me? So, I decided to drink …

Nicole

Nicole's account further shows how proximity to her abuser intensified unresolved anger and avoidance. Her resort to alcohol demonstrates an attempt to regain control over unbearable emotions, a meaning‐making process that frames drinking as both coping and escape.

When the abuse started again, it broke me again. […] I remember my friend whose boyfriend was selling rocks, but this time I was thinking this is going to bring me into deep water if I do this (use crack cocaine) but, at the same time, I was thinking that I needed this.

Megan

Megan's reflection captures the tension between awareness and necessity. Her words reveal the conscious negotiation between self‐destruction and relief, demonstrating how trauma reshapes agency within constrained contexts. Substance use becomes a rationalized strategy for emotional survival.

#### 2.7.2 Substance use Related to Other Adverse Life Experiences

Substance use was associated not only with experiences of victimization but also with other adverse life experiences. These experiences included the death of a loved one, feelings of disconnection, exposure to caregivers' substance use, and peer influence regarding substance use.

##### 2.7.2.1 Loss of a Loved One

The loss of a loved one was frequently cited as a contributing factor to substance use. For some women, this loss marked a significant turning point in their lives. Often, this was the death of a close caregiver, such as a parent or guardian. This profound loss contributed to feelings of isolation and a lack of social support, leading some women to use substances as a coping mechanism.

I was 16, and she [mum] is the person who was supportive towards me. She never abandoned me. After Mum's death, that's where my life started to lack. When I say “lack” I mean the full realization that I was alone, and I felt it even in my being that something had changed because I didn't have anyone to cry to. […] Yes, I did drink alcohol and I would get enraged when I talk about these things. I used to drink alcohol and used drugs.

Emily

Emily's narrative conveys how maternal loss generated an enduring sense of emptiness. Substance use emerged as a way to mediate grief and sustain a connection to her late mother through emotional release.

Sadly, he (my uncle) passed in 2011, and I was left with my aunt. Then, my aunt started changing up on me.

Anna

Anna's reflection illustrates how bereavement disrupted her family stability. The emotional neglect that followed prompted substance use as a means of regaining comfort and control.

##### 2.7.2.2 Feelings of Disconnection

Several women described experiencing neglect from close family members, including parents or guardians. This led them to seek a sense of belonging and connection outside the family. These feelings of disconnection contributed to their substance use.

I never had a real family, so I always just felt alone; I did not feel my own family cared for me.

Jessica

Jessica's statement relays how perceived emotional abandonment can heighten loneliness. Her words demonstrate how isolation shaped the meaning she attached to substance use as comfort in the absence of care.

I know my mother did not love me. […] I kind of blame my mother (for the substance use) because I keep thinking had she accepted me, I would not be here (in the correctional center). I have given up and I have made peace that she does not love me. My mother loves other people's children, takes care of them and cooks for them.

Ashley

Ashley's reflection portrays substance use as a response to maternal rejection. Her meaning‐making highlights how the absence of maternal affection can contribute to self‐blame and emotional withdrawal.

I was at the age of 17. No one was there for me, so I decided to just go my own way. […] What was painful is that it was not just one guy (rape perpetrator), it was many. My father was supposed to be supportive, but he also did not do anything.

Patricia

Patricia's experience shows how cumulative betrayal by both perpetrators and passive caregivers deepened her disconnection. Substance use, in this context, symbolized an effort to manage unresolved anger and abandonment.

##### 2.7.2.3 Exposure to Caregivers' Substance use

As previously noted, children exposed to substance use by their parents or caregivers are more likely to experience substance use challenges themselves. Several participants described witnessing substance use within their families.

He (father) would drink normally after work. […] With the drinking, I used to think that's the way things are supposed to be.

Kimberly

Kimberly's observation shows how normalized patterns of drinking within the family created an implicit model for adult coping.

My mother and my father used to drink alcohol a lot. They would drink every day; there was no special occasion for them.

Mary

Mary's description demonstrates the intergenerational transmission of substance use norms where daily drinking was perceived as routine family behavior.

They used to smoke dagga. It was my mum and my uncle and another cousin.

Jennifer

Jennifer's account reflects early exposure to substance use as an accepted social activity, shaping her later attitudes toward substance use.

My mother was an alcoholic.

Samantha

Samantha's brief but direct statement encapsulates how parental dependency often coexists with emotional absence, influencing children's future vulnerabilities.

I was 18 years and this one lady used to come to clean the house. I used to catch her so many times doing drugs. This one time, I found her lolly (paraphernalia for using substances) with the lighter there. I left it for a few months. […] But that day something just said light this thing and I did.

Melissa

Melissa's narrative reveals how curiosity and proximity to others' drug use can initiate experimentation, particularly when supervision or guidance is lacking.

Caregivers' substance use often coincided with an absence of parental figures or adequate supervision. Substance use within a family can create financial strain as resources are diverted to support substance dependence. This can contribute to children's feelings of neglect. In some cases, children subsequently began using substances as a way to cope with these circumstances.

I didn't have a mother figure, so my father was my mother and my father. So, when my mother came back, I couldn't do it. She wanted to drink, and she wanted me to go to the family to go look for money. I would get the money from the family, but she would buy alcohol with it. […] My family wanted to put me in a job, which I didn't want and that's when I ran away from my family.

Jessica

Jessica's reflection illustrates how exposure to parental alcohol use created resentment and escape behaviors. Her decision to leave home demonstrates agency formed through rejection of unhealthy family patterns.

##### 2.7.2.4 Peer and Environmental Influences on Substance use

Many participants reported that their initial exposure to substances was through peer interactions. This influence was often intertwined with experiences of victimization and other adverse life events. Some participants described being simultaneously influenced by peer dynamics and their surrounding environment, which usually included subcultures where substance use was normalized.

I grew up on the Cape Flats. At the Cape, there is this type of lifestyle; you are either in or out. When you are out, you become the popeye (not taken seriously), and nobody wants to be the popeye. So, me and my friends did drugs.

Kimberly

Kimberly's words show how belonging and identity formation within peer culture can normalize drug use as a marker of social acceptance.

So, I started working at a textile factory at 14. […] So, when I started working I met friends and with the friends I got introduced to drugs. […] So, I had more time to roam around with friends. I met up with friends and they were up to this and that, and they introduced me to dagga (marijuana) and mandrax (methaqualone).

Jessica

Jessica's account links early workforce participation and peer influence to exposure to substances, showing how autonomy without guidance increased vulnerability.

I started (drug use) at the age of 17 with my friends. From a young age, they were already smokers. They would always tell me to try it out. But I have a serious heart problem, so I was always scared. Until one day I was just tired of them, so I said let me just try.

Stephanie

Stephanie's reflection captures peer pressure as a catalyst for initiation into substance use, highlighting the interplay between curiosity, fatigue, and conformity within adolescent networks.

#### 2.7.3 Association Between Substance use and Involvement With the Criminal Justice System

Most of the women in this study reported not having formal employment. They generally came from lower socioeconomic backgrounds and had limited educational opportunities. Consequently, they often engaged in activities that brought them into contact with the CJS to obtain money for substances. These activities primarily involved property‐related offenses such as theft and, in some cases, involvement in drug distribution. Women who had informal employment sometimes used earnings from these jobs to purchase substances. However, when they were unable to maintain their employment, some resorted to property‐related offenses. Other participants described obtaining money for substances through the deception of family members.

Now, in 2020, that's when I started stealing. I was desperate, I needed to help myself to survive. I'd go to people's houses, usually big houses, and I'd steal phones, irons, kettles, groceries, wallets, and money. […] After stealing I'd buy food, drugs, cold drinks, and chips.

Brittany

Brittany's account portrays survival‐driven offending linked to economic marginalization. Substance use and theft appear intertwined as adaptive strategies for coping with poverty and dependency.

I was taking ecstasy practically every weekend and I was dealing it in the clubs as well. Then I would find myself taking money from the petty cash at work so I can support my habit.

Amanda

Amanda's reflection demonstrates how dependence on substances blurred moral and economic boundaries. Her actions illustrate how addiction and financial pressure intersect in environments that normalize recreational drug use.

I always had money, because I was working, but it was never enough. You become greedy. The amount that you use just keeps on increasing … As I was saying, the money is never enough. I was never a person who could break into people's houses or rob them, my justification was that I would rather walk into a shop where I did not know the owner, and there is enough, so they would not notice if I took something. That was my justification. You end up stealing then go sell it.

Pearl

Pearl's account reveals cognitive rationalization as a coping mechanism for morally conflicting behavior. Her reasoning highlights how addiction can distort ethical boundaries while preserving a sense of self‐justification.

…my parents would also give me money sometimes when I ask. My sister is the one who always gives me money when I ask.

Stephanie

Stephanie's statement illustrates the role of familial support in sustaining dependency. Access to money within family networks inadvertently enabled ongoing substance use, demonstrating how relational dynamics can reinforce cycles of dependence.

## 3 Discussion

These narratives support the established association between women's experiences of victimization and substance use (Clark and Vella 2022; Liu et al. 2021; Rivas‐Rivero et al. 2020), thus expanding them to a less‐studied South African context. The analysis also highlighted an association between substance use and involvement with the CJS. It is important to note that many previous studies examining the link between substance use and CJS involvement primarily focused on male participants (French et al. 2000; Sharma 2022; Simpson 2003). This study, centered on women, also identified participants' accounts suggesting this association. One distinction between this study and male‐focused studies pertains to the differing motivations often associated with substance use. Research on men commonly links substance use to peer dynamics, criminal subcultures, and economic motives such as profit‐driven drug markets (French et al. 2000; Simpson 2003). In contrast, the women in this study described substance use as a coping response to victimization, trauma, and adverse life experiences, highlighting gender‐specific pathways into offending. The analysis suggested that adverse life experiences beyond victimization, such as the loss of a parent or neglect from caregivers, were associated with women's substance use. This finding aligns with Rivas‐Rivero et al.'s (2020) analysis of women's frequent patterns of substance use in relation to stressful life events, including economic problems and victimization. Exposure to caregivers' substance use also often coincided with reduced supervision, increasing vulnerability to peer influence and substance use. These insights are consistent with other studies, conducted in the United States and United Kingdom, examining women's criminal behavior and substance use (Leban and Gibson 2020; Mitchell et al. 2022; Peterson et al. 2019; Saxena and Messina 2021).

### 3.1 Connecting to CP Theory: Ecological Systems Theory

This analysis of adverse life experiences contributes to the growing body of knowledge in the area of CP, highlighting the direct and indirect pathways through which adverse experiences can contribute to substance use and behaviors that bring women into contact with the CJS. These meanings can be understood through Bronfenbrenner's (1979) Ecological Systems Theory. The loss of a parent or neglect from caregivers directly impacts the *microsystem* (the immediate family environment) and disrupts the individual's primary support system. Exposure to caregiver substance use further destabilizes the microsystem and can negatively influence the *mesosystem* (the interactions between the family and other systems, such as schools or peer groups). For instance, children exposed to caregiver substance use may experience irregular school attendance, strained teacher–parent relationships, or peer rejection, thereby weakening the supportive links between family and educational settings that constitute the mesosystem. These experiences prompted the women in this study to seek coping mechanisms, with substance use serving as one such mechanism. This finding supports existing literature indicating a connection between women's substance use and coping with psychological distress (Asberg and Renk 2012, 7; Bayliss 2016, 28–37; Baltieri 2014, 123; Heilemann and Santhiveeran 2011, 70). As the narratives suggest, substances were used to attempt to escape the psychological distress of life's adversities. However, substances do not provide a long‐term solution, as psychological distress often returns, sometimes with increased intensity, after the effects of the substance subside (Jamatia 2023, 70; Dye 2018, 384). This cycle can contribute to the development of substance dependence. Specific triggers for substance use among the women in this study included physical and emotional abuse from partners, feelings of rejection from family members, and experiences of sexual assault in childhood. These interpretations align with existing knowledge that highlight the established link between incarcerated women and experiences of physical and sexual victimization (Green et al. 2016, 458–459; Karlsson and Zielinski 2020, 332–333; Tripodi and Pettus‐Davis 2013, 33–34).

### 3.2 Connecting to CP Theory: Social Justice/Liberation Psychology

From a social justice perspective, these interpretations highlight the need to address the systemic inequalities that contribute to women's vulnerability to victimization, substance use, and incarceration. The disproportionate impact of adverse life experiences on marginalized communities, often exacerbated by factors such as poverty, lack of access to resources, and historical trauma related to apartheid (Ratele and Malherbe 2022), speaks to the need for systemic change. Liberation Psychology (Martín‐Baró 1994) further emphasizes the importance of understanding the psychological consequences of oppression and empowering marginalized communities to challenge oppressive systems.

### 3.3 Behavioral Coping Strategies and CP: Sense of Community/Social Support

Behavioral coping strategies can be categorized into two types: (1) actions aimed at reducing or eliminating the source of psychological distress and (2) actions focused on obtaining alternative rewards, such as theft, as a means of managing psychological distress (Stallman 2020, 296). This study indicated that the women participants engaged in both types of coping strategies, often concurrently. They experienced psychological distress and subsequently used substances to alleviate the associated negative feelings. However, substance use often led to further distress, such as withdrawal symptoms or the return of stressors once the effects of the substances wore off. To obtain more substances to manage the renewed stressors, some participants engaged in activities that brought them into contact with the CJS. Thus, these two coping strategies interacted to contribute to the complex relationship between substance use and CJS involvement observed in this study. From a CP perspective, the lack of strong social support networks and a sense of community (Sarason et al. 1990) can significantly contribute to individuals' reliance on maladaptive coping mechanisms like substance use. This study highlighted the participants' limited access to support structures and the challenges they faced in developing effective coping skills.

### 3.4 Recommendations and Implications for CP Practice and Action

Drawing from these interpretations, several recommendations can be made to address the complex interplay of childhood adversity, substance use, and involvement with the CJS among women. From a CP perspective, interventions should focus on multiple levels of intervention, addressing both individual needs and systemic issues. Understanding the impact of childhood adversities on substance use and subsequent involvement with the CJS enables the development of more effective intervention strategies at various stages of girls' and women's lives. The loss of a loved one, for example, is a critical juncture that signals a need for strong support systems to prevent substance use as a coping mechanism. The presence of illicit substances in the home and the introduction of alcohol or other substances to minors should be addressed through preventive and family‐focused interventions implemented jointly by justice and social service systems. Positive parental and caregiver involvement plays a vital role in safeguarding girls and young women within and outside the family context. Public awareness campaigns should emphasize the impact of adverse life experiences, promote positive parenting practices, and encourage the creation of healthy, supportive home environments. These interpretations also highlight the unique challenges faced by girls and women who experience multiple traumas, particularly victimization. Targeted resources should be provided to professionals working with younger and older women who have experienced various forms of abuse, co‐occurring disorders, or who require trauma‐informed care.

To that effect, there is a need for specific CP‐informed interventions. Intervention programs for girls and young women exposed to adversities should focus on disrupting the link between these experiences and subsequent substance use or involvement with the CJS. These programs should aim to address the root causes of these challenges and establish support systems to prevent further involvement. From a CP perspective, this means developing empowering, community‐based interventions that address the social and structural determinants of health. Examples include:

- Trauma‐informed care: Providing trauma‐informed services within correctional facilities and community settings that recognize the impact of trauma on women's lives and promote healing and recovery.
- Empowerment programs: Implementing programs that build women's self‐efficacy, promote leadership skills, and provide opportunities for social and economic empowerment.
- Community‐based support groups: Establishing peer support groups and community networks where women can connect with others who have shared similar experiences and receive emotional support and practical assistance.
- Advocacy and policy change: Advocacy efforts should focus on addressing systemic inequalities, such as poverty and lack of affordable housing. Policy changes should also prioritize increasing access to education and employment opportunities. This aligns with CP's focus on social justice and systems change.

### 3.5 Future Research and Implications for the Field of CP

Future research should explore women's experiences with substance use in more nuanced and intersectional ways. While existing research on factors contributing to women's substance use and involvement with the CJS is valuable, it often does not fully capture the complexities of individual experiences or the impact of social inequalities. In particular, there remains limited empirical research on women's substance use and CJS involvement within the South African context, highlighting an important area for future investigation. Therefore, future studies should adopt an intersectional lens, recognizing the interplay of multiple identities (e.g., race, ethnicity, gender, class, religion, language, income, occupation, and sexuality) in shaping individual experiences. Future research could include individuals in substance use treatment programs. Additionally, it should consider those detained but not yet tried or sentenced to provide a broader understanding of experiences across various stages of involvement with the CJS. Additionally, quantitative research is needed to investigate the role of substance use as a mediating or moderating variable in the relationship between childhood trauma and adult involvement with the CJS. This would further clarify the complex relationships between these factors and inform the development of more effective interventions. This study contributes to the field of CP by highlighting the need for contextually relevant and empowering interventions that address the social and structural determinants of women's involvement in the CJS. This study highlights a crucial need for the field to further integrate understandings of trauma, substance use, and systemic oppression in interventions and policy recommendations for women involved in the CJS.

## 4 Limitations

This study has several limitations. First, although this approach emphasizes in‐depth understanding, the use of nonprobability sampling limits the transferability of insights to other populations of women who have had contact with the CJS. Second, while the study relied on self‐reported data, the concern about recall bias is less applicable in this interpretivist design, as the focus is on how participants construct meaning from their experiences rather than on the objective accuracy of their recollections. A further limitation concerns the exclusive focus on cisgender women. This focus was not an explicit exclusion criterion but rather a result of the snowball sampling approach. The approach did not yield participants identifying as transgender or gender diverse. The absence of these perspectives limits understanding of how gender diversity might shape experiences of substance use and CJS involvement. Future research should purposefully include gender‐diverse participants to provide a more comprehensive account of experiences across gender identities.

## 5 Conclusion

This study, viewed through a CP lens, highlights how childhood trauma, victimization, and systemic inequalities shape women's experiences with substance use and CJS involvement. The analysis emphasizes the need for interventions that address the root causes of these challenges, including poverty, lack of access to resources, and the enduring legacies of historical trauma. Addressing these issues requires a multilevel approach that empowers women, strengthens community support systems, and advocates for social justice. Future research should prioritize inclusivity and explore the experiences of diverse gender identities. Addressing these limitations and expanding our understanding of the diverse experiences of women within the CJS will enable the development of more effective, equitable, and community‐based interventions that promote healing, recovery, and social justice.

## Conflicts of Interest

The author declares no conflicts of interest.

## Data Availability

The data that support the findings of this study are available on request from the corresponding author. The data are not publicly available due to privacy or ethical restrictions.
